# Supplementary figures and images for: Association between PDE4D rs966221 and the Risk of Ischemic Stroke in Regional Chinese Populations
Source: Brain Sci. 2023 Jul 7;13(7):1038. doi: 10.3390/brainsci13071038 (PMC10377348; doi:10.3390/brainsci13071038)

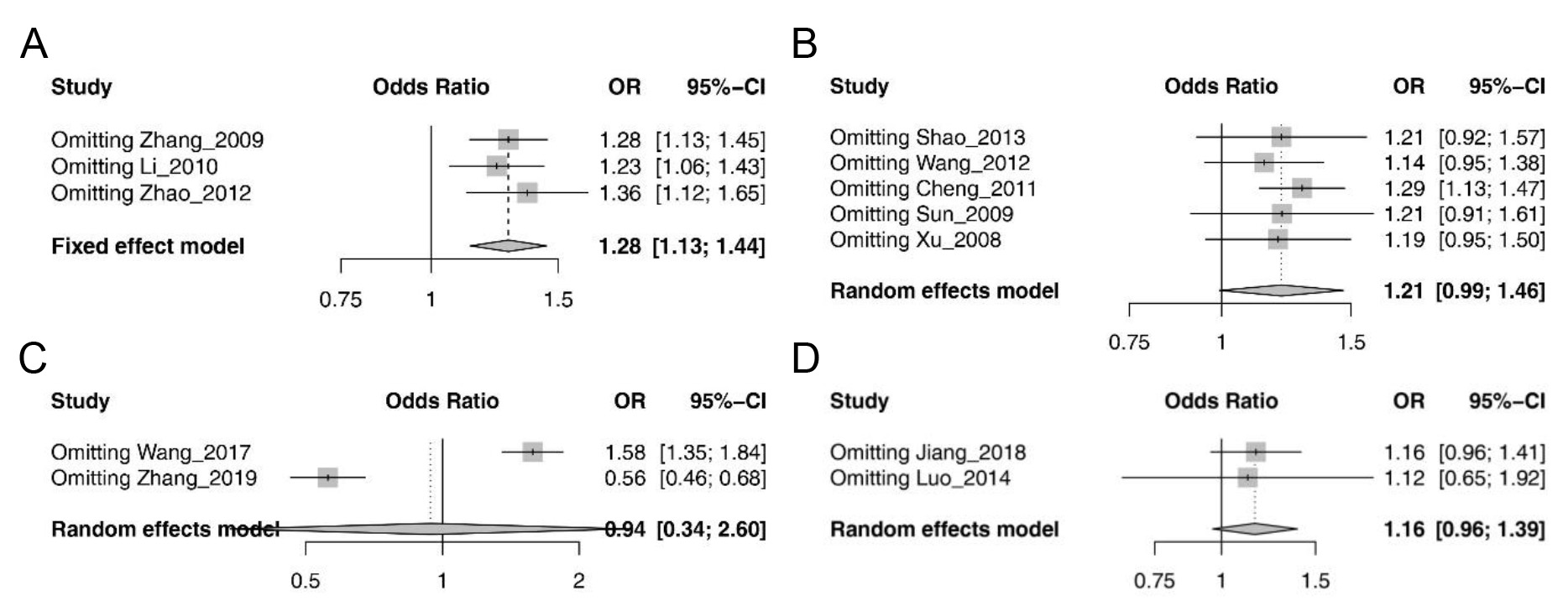

Supplement: Supplementary file 1 [file brainsci-13-01038-s001.zip › brainsci-2411777-supplementary/brainsci-2411777-supplementary-Figure S1 Forest plot of relationship between rs966221 and IS.jpg]
